# Implementation research: including breast examinations in a cervical cancer screening programme, Rwanda

**DOI:** 10.2471/BLT.22.289599

**Published:** 2023-05-26

**Authors:** Lydia E Pace, Marc Hagenimana, Jean-Marie Vianney Dusengimana, Jean Paul Balinda, Origene Benewe, Vestine Rugema, Jean de Dieu Uwihaye, Amanda Fata, Cyprien Shyirambere, Lawrence N Shulman, Nancy L Keating, Francois Uwinkindi

**Affiliations:** a Division of Women’s Health, Department of Medicine, Brigham and Women’s Hospital, 75 Francis Street, Boston, MA 02115, United States of America (USA).; b Rwanda Biomedical Centre, Kigali, Rwanda.; c Partners In Health, Kigali, Rwanda.; d Rwanda Ministry of Health, Burera District, Rwanda.; e Abramson Cancer Center, University of Pennsylvania, Philadelphia, USA.; f Department of Health Care Policy, Harvard Medical School, Boston, USA.

## Abstract

**Objective:**

To evaluate whether integrating breast and cervical cancer screening in Rwanda’s Women’s Cancer Early Detection Program led to early breast cancer diagnoses in asymptomatic women.

**Methods:**

Launched in three districts in 2018–2019, the early detection programme offered clinical breast examination screening for all women receiving cervical cancer screening, and diagnostic breast examination for women with breast cancer symptoms. Women with abnormal breast examinations were referred to district hospitals and then to referral hospitals if needed. We examined how often clinics were held, patient volumes and number of referrals. We also examined intervals between referrals and visits to the next care level and, among women diagnosed with cancer, their initial reasons for seeking care.

**Findings:**

Health centres held clinics > 68% of the weeks. Overall, 9763 women received cervical cancer screening and clinical breast examination and 7616 received breast examination alone. Of 585 women referred from health centres, 436 (74.5%) visited the district hospital after a median of 9 days (interquartile range, IQR: 3–19). Of 200 women referred to referral hospitals, 179 (89.5%) attended after a median of 11 days (IQR: 4–18). Of 29 women diagnosed with breast cancer, 19 were ≥ 50 years and 23 had stage III or stage IV disease. All women with breast cancer whose reasons for seeking care were known (23 women) had experienced breast cancer symptoms.

**Conclusion:**

In the short-term, integrating clinical breast examination with cervical cancer screening was not associated with detection of early-stage breast cancer among asymptomatic women. Priority should be given to encouraging women to seek timely care for symptoms.

## Résumé

**Objectif:**

Évaluer si le fait d’intégrer le dépistage du cancer du sein et du col de l’utérus dans le Programme de détection précoce du cancer chez les femmes au Rwanda a permis de mieux diagnostiquer les cancers du sein chez les femmes asymptomatiques.

**Méthodes:**

Lancé dans trois districts en 2018-2019, le programme de détection précoce proposait un examen clinique des seins à toutes les femmes effectuant un dépistage du cancer du col de l'utérus et un examen diagnostique des seins à celles présentant des symptômes de cancer du sein. Les femmes ayant obtenu un résultat anormal lors de cet examen étaient ensuite transférées à des hôpitaux de district et, si nécessaire, à des hôpitaux de référence. Nous nous sommes intéressés à la fréquence à laquelle les cliniques ont été mises sur pied, au volume de patients et au nombre de transferts. Nous avons également analysé les intervalles entre les transferts et les visites au niveau de soins supérieur et, pour les femmes atteintes d'un cancer, les raisons initiales qui les ont incitées à consulter.

**Résultats:**

Les centres de santé ont organisé des cliniques > 68% des semaines. De manière générale, 9763 femmes ont été soumises à un dépistage du cancer du col de l'utérus et à un examen clinique des seins, tandis que 7616 d'entre elles n'ont fait l'objet que d'un examen des seins. Sur les 585 femmes transférées depuis les centres de santé, 436 (74,5%) se sont rendues dans l'hôpital de district après un délai médian de neuf jours (écart interquartile, EI: 3-19). Sur les 200 femmes renvoyées vers des hôpitaux de référence, 179 (89,5%) s'y sont rendues après un délai médian de 11 jours (EI: 4-18). Enfin, sur les 29 femmes ayant reçu un diagnostic de cancer du sein, 19 étaient âgées de 50 ans et plus, et 23 souffraient d'un cancer de stade III ou IV. Toutes les femmes atteintes d'un cancer du sein ayant partagé les raisons qui les ont poussées à consulter (23 femmes) avaient ressenti des symptômes de la maladie.

**Conclusion:**

À court terme, intégrer un examen clinique du sein lors d'un dépistage du cancer du col de l'utérus n’a pas donné lieu à une détection précoce du cancer du sein chez les femmes asymptomatiques. La priorité est d'encourager les femmes à consulter rapidement dès l'apparition de symptômes.

## Resumen

**Objetivo:**

Evaluar si la integración de la detección del cáncer de mama y de cuello uterino en el Programa de Detección Temprana del Cáncer en Mujeres de Ruanda permitió realizar diagnósticos tempranos de cáncer de mama en mujeres asintomáticas.

**Métodos:**

Lanzado en tres distritos entre 2018 y 2019, el programa de detección temprana ofrecía exámenes clínicos de mama a todas las mujeres que se sometían al cribado del cáncer de cuello uterino y exámenes mamarios diagnósticos a las mujeres con síntomas de cáncer de mama. Las mujeres con exámenes de mama anormales eran derivadas a hospitales de distrito y, posteriormente, a hospitales de referencia si era necesario. Se examinó la frecuencia de las consultas, el volumen de pacientes y el número de derivaciones. También se examinaron los intervalos entre las derivaciones y las visitas al siguiente nivel asistencial y, entre las mujeres diagnosticadas de cáncer, sus motivos iniciales para buscar asistencia.

**Resultados:**

Los centros sanitarios realizaron consultas >68% de las semanas. En total, 9763 mujeres se sometieron a detección del cáncer de cuello uterino y examen clínico de las mamas, y 7616 solo a examen de las mamas. De 585 mujeres remitidas desde los centros sanitarios, 436 (74,5%) acudieron al hospital de distrito tras una mediana de 9 días (rango intercuartílico, IQR: 3-19). De 200 mujeres remitidas a hospitales de referencia, 179 (89,5%) acudieron tras una mediana de 11 días (IQR: 4-18). De 29 mujeres diagnosticadas de cáncer de mama, 19 tenían ≥50 años y 23 presentaban enfermedad en estadio III o estadio IV. Todas las mujeres con cáncer de mama, cuyos motivos de búsqueda de atención eran conocidos (23 mujeres) habían experimentado síntomas de cáncer de mama.

**Conclusión:**

A corto plazo, la integración del examen clínico de las mamas con la detección del cáncer de cuello uterino no se asoció con la detección temprana del cáncer de mama entre las mujeres asintomáticas. Debe darse prioridad a animar a las mujeres a buscar atención oportuna ante los síntomas.

## ملخص

**الغرض:**

تقييم ما إذا كان دمج فحص سرطان الثدي وسرطان عنق الرحم في برنامج الكشف المبكر عن السرطان لدى النساء في رواندا، قد أدى إلى التشخيص المبكر لسرطان الثدي لدى النساء اللاتي لا تظهر عليهن الأعراض.

الطريقة **إن برنامج ** الكشف المبكر، الذي تم إطلاقه في ثلاث مقاطعات خلال الفترة 2018 إلى 2019، قدم لجميع النساء اللواتي يخضعن لفحص سرطان عنق الرحم، وفحص الثدي التشخيصي للنساء المصابات بأعراض سرطان الثدي. تمت إحالة النساء اللواتي لديهن فحوصات غير طبيعية للثدي إلى مستشفيات المقاطعات، ثم إلى مستشفيات الإحالة إذا لزم الأمر. قمنا بفحص عدد المرات التي عُقدت فيها العيادات، وأعداد المرضى، وعدد الإحالات. قمنا أيضًا بفحص الفترات الفاصلة بين الإحالات والزيارات إلى مستوى الرعاية التالي، وفحص الأسباب الأولية لطلب الرعاية بين النساء المصابات بالسرطان.

**النتائج:**

أقامت المراكز الصحية عيادات خلال أكثر من %68 من الأسابيع. إجمالاً، خضعت 9763 امرأة لفحص سرطان عنق الرحم، وفحص إكلينيكي للثدي، وخضعت 7616 امرأة لفحص الثدي وحده. من بين 585 امرأة تمت إحالتهن من المراكز الصحية، زارت 436 (%74.5) منهن مستشفى المنطقة بعد متوسط 9 أيام (المدى بين الشرائح الربيعية (IQR): 3 إلى 19). من بين 200 امرأة تمت إحالتهن إلى مستشفيات الإحالة، حضرت 179 (%89.5) منهن بعد متوسط 11 يومًا (المدى بين الشرائح الربيعية: 4 إلى 18). من بين 29 امرأة تم تشخيص إصابتهن بسرطان الثدي، كانت 19 منهن بسن 50 سنة أو أكثر، و23 منهن مصابات بالمرحلة الثالثة، أو المرحلة الرابعة من المرض. جميع النساء المصابات بسرطان الثدي، اللواتي كانت أسباب طلبهن للرعاية معروفة (23 امرأة)، عانين من أعراض سرطان الثدي.

**الاستنتاج:**

إن دمج فحص الثدي الإكلينيكي مع فحص سرطان عنق الرحم، لم يرتبط بالكشف عن سرطان الثدي في المراحل المبكرة لدى النساء اللاتي لا تظهر عليهن الأعراض. يجب إعطاء الأولوية لتشجيع النساء على طلب الرعاية في الوقت المناسب للأعراض.

## 摘要

**目标:**

旨在评估 “卢旺达妇科癌症早期检出项目” 将乳腺癌和宫颈癌筛查相结合是否有利于无症状妇女的乳腺癌早期诊断。

**方法:**

早期检出项目于 2018-2019 年在三个地区开展，为所有接受宫颈癌筛查的妇女提供了临床乳房检查，并为有乳腺癌症状的妇女提供诊断性乳房检查。乳房检查异常的妇女被转诊到地区医院，如果需要，再转诊到转诊医院。我们调查了诊所的设立频率、患者数量和转诊人数。我们还调查了确诊为癌症的妇女在转诊和进一步寻求治疗期间的时间间隔，以及她们寻求治疗的最初原因。

**结果:**

在此期间，卫生中心设立诊所的的时间超过 68%。总体而言，9,763 名妇女接受了宫颈癌筛查及临床乳房检查，7,616 名妇女只接受了乳房检查。在从卫生中心转诊的 585 名妇女中，436 (74.5%) 名妇女在中位数 9 天（四分位距，IQR：3-19）后去了地区医院就诊。在 200 名转诊到转诊医院的妇女中，179 名 (89.5%) 妇女在中位数 11 天(IQR: 4-18）后去了地区医院就诊。在 29 名被诊断为乳腺癌的妇女中，19 名年龄 ≥ 50 岁，23 名患有 III 期或 IV 期疾病。所有寻求治疗原因已知的乳腺癌女性患者（23 名妇女）均已出现乳腺癌症状。

**结论:**

短期内，将临床乳腺检查与宫颈癌筛查相结合与无症状妇女早乳腺癌的诊断并无关联。应优先鼓励妇女在出现症状时及时寻求治疗。

## Резюме

**Цель:**

Оценить, способствовало ли объединение скрининга на рак молочной железы и на рак шейки матки в ходе программы раннего выявления рака у женщин в Руанде выявлению рака молочной железы на ранних стадиях у бессимптомных женщин.

**Методы:**

В рамках программы раннего выявления, запущенной в трех районах страны в период с 2016 по 2018 годы, всем женщинам, прошедшим скрининг на рак шейки матки, предлагалось пройти клиническое обследование молочных желез, а женщинам с симптомами рака молочной железы – диагностическое обследование молочных желез. Женщин с выявленными при обследовании молочных желез отклонениями направляли в районные больницы, а затем при необходимости в лечебно-диагностические центры. Были изучены такие факторы, как частота проведения консультаций, количество пациентов и количество направлений к врачу. Также изучались интервалы между выдачей направлений к врачу и визитами в медицинские учреждения следующего уровня и среди женщин с установленным диагнозом рака их первоначальные причины обращения за медицинской помощью.

**Результаты:**

В центрах здравоохранения проводились консультации на протяжении более 68% недель. В целом 9763 женщины прошли скрининг на рак шейки матки и клиническое обследование молочных желез, а 7616 – только обследование молочных желез. Из 585 женщин, направленных из центров здравоохранения, 436 (74,5%) обратились в районную больницу в среднем через 9 дней (межквартильный интервал, IQR: 3–19). Из 200 женщин, направленных в лечебно-диагностические центры, 179 (89,5%) были госпитализированы через 11 дней (IQR: 4–18). Из 29 женщин с установленным диагнозом рака молочной железы 19 находились в возрасте ≥ 50 лет, а у 23 была III или IV стадия заболевания. У всех женщин с диагнозом рака молочной железы, причины обращения за медицинской помощью которых были известны (23 женщины), наблюдались симптомы заболевания.

**Вывод:**

В краткосрочной перспективе объединение клинического обследования молочных желез со скринингом на рак шейки матки не ассоциировалось с выявлением ранних стадий рака молочной железы у бессимптомных женщин. В первую очередь следует призывать женщин своевременно обращаться за медицинской помощью при появлении симптомов.

## Introduction

Breast cancer incidence is increasing rapidly in sub-Saharan Africa. Even though the age-standardized incidence rates of breast cancer in sub-Saharan Africa are still lower than in higher-income regions, women in sub-Saharan Africa face a greater likelihood of dying of breast cancer, largely because of long delays in diagnosis and late-stage diagnosis.^1^^,^^2^ Strategies to facilitate earlier breast cancer detection are crucial to improving breast cancer outcomes in sub-Saharan Africa.

While mammography is the breast cancer screening strategy most supported by scientific evidence,^3^ it is costly and not yet feasible in most low- and middle-income countries, including in sub-Saharan Africa.^4^^,^^5^ Mammography screening is also less effective in women younger than 50 years, so other strategies may prove to be more beneficial for sub-Saharan Africa’s younger population.^6^ Studies show that in countries where mammography screening is not yet available, clinical breast examination screening leads to diagnosis of breast cancer at earlier stages.^7^^–^^10^ Clinical breast examination screening may also reduce breast cancer mortality in women older than 50 years.^11^ High-quality diagnostic clinical breast examination is essential in evaluating individuals with breast symptoms in any setting.

No consensus exists about how to effectively integrate early detection of breast cancer into resource-constrained primary health-care systems in low- and middle-income countries. The Global Breast Cancer Initiative of the World Health Organization (WHO) and the Breast Health Global Initiative emphasize that expediting diagnosis in women with breast cancer symptoms is a crucial first step in early detection.^12^^,^^13^ Approaches to facilitate early diagnosis include increased community awareness; timely recognition of symptoms; high-quality diagnostic clinical breast examination; and prompt referral for imaging and biopsy.^12^^–^^15^ In settings where most breast cancers are diagnosed at a late stage, the WHO Global Breast Cancer Initiative notes that identifying symptomatic cancers earlier is likely to result in important shifts in cancer stage at diagnosis and reduced breast cancer mortality, at a lower cost than screening of asymptomatic individuals.^16^ However, limited empirical evidence is available on the trade-offs between an early diagnosis approach (focusing on individuals with breast cancer symptoms) versus clinical breast examination screening (targeting a broader population) in a resource-constrained environment. To enhance the reach and feasibility of early detection of breast cancer, there is interest in integrating breast cancer screening with cervical cancer screening, given the established benefit of cervical cancer screening and the potential overlap in target populations and messages.^17^^–^^19^ However, evidence is lacking to guide implementation of this integrated approach.

Rwanda is a small, densely populated East African country of 13 million people designated as low-income by the World Bank.^20^ Breast cancer is the most commonly diagnosed cancer at the Butaro Cancer Center of Excellence, Rwanda’s public cancer referral facility. Three in four women with breast cancer attending the centre are diagnosed at stage III or stage IV,^21^ when breast cancer is either difficult or impossible to cure. In 2012–2014, the delays Rwandan women experienced between the onset of symptoms and breast cancer diagnosis were among the longest reported in the literature.^2^ To reduce these delays, in 2015 clinicians, public health leaders, and researchers from Rwanda’s Ministry of Health; Rwanda’s national health implementation agency (Rwanda Biomedical Centre); the nongovernmental organization Partners In Health; the Brigham and Women’s Hospital, United States of America (USA); and the University of Pennsylvania, USA, launched a pilot programme for early diagnosis of breast cancer in Burera District. This programme focused on facilitating timely diagnosis in women with breast cancer symptoms by building capacity at multiple levels of the heath system. The programme improved the knowledge and skills of community health workers (CHWs) and nurses,^22^ increased visits by women for breast concerns and increased early-stage cancer diagnoses.^23^ The cost of clinical breast examination at health centres was about 3.27 United States dollars (US$) per visit, comparable to other primary care services. Over 2 years, the programme cost about US$ 1482.00 per breast cancer diagnosed.^24^

Based on the success of the Burera pilot programme, Rwanda Biomedical Centre leadership sought to build primary care capacity to scale up early detection of breast cancer. During planning meetings, clinical stakeholders observed that while the Burera pilot programme was effective, start-up costs were high and stand-alone breast clinics were inefficient. Interest was expressed in integrating early detection of breast cancer with cervical cancer screening to more efficiently use training opportunities, staff time, space and community outreach, and jointly address the two most common cancers in Rwandan women, as prioritized in Rwanda’s National Cancer Control Plan.^25^ Given low breast cancer awareness in the community, stakeholders were also concerned about relying on women seeking care for breast cancer symptoms. As a means to increase detection of asymptomatic breast cancers, they recommended clinical breast examination screening for all women receiving cervical cancer screening, regardless of whether they had breast cancer symptoms or not. Rwanda Biomedical Centre incorporated this input into a combined breast and cervical cancer initiative, the Women’s Cancer Early Detection Program. The programme aimed to reach more women and improve early cancer detection and prevention through integrating breast and cervical cancer screening. The programme was initially launched in three Rwandan districts in 2018–2019.

To evaluate programme implementation and outcomes, programme leaders and partnering researchers gathered clinical and programme data from July 2018 to December 2019. In particular, we sought to understand whether adding clinical breast examination screening to cervical cancer screening led to early diagnoses of breast cancer in asymptomatic women that would not have been identified through engagement with symptomatic women only. Our goal was to guide further scale-up of the programme in Rwanda, and provide information for other countries seeking to integrate early detection of breast cancer into other clinical initiatives.

## Methods

### Study design and setting

We conducted a retrospective observational study of the early detection programme to examine programme operations and patient outcomes. The three districts where the programme was launched were Rwamagana, Rubavu and Kirehe which together have 42 health centres, three district hospitals and covering 1.3 million people.

### Participants

All women eligible for cervical cancer screening – aged 30–49 years, based on Rwanda’s and WHO’s policies at the time^26^ – also received clinical breast examination screening. In addition, women with breast concerns of any age were invited to receive a diagnostic clinical breast examination.

### Programme implementation

Implementation of the programme started with a half-day training session for CHWs, and a 2-week on-site training session (1 week of teaching and 1 week of supervised practice) for all clinicians at the health centres and hospitals. These training sessions included cervical cancer screening as well as the breast health curriculum used in the Burera pilot programme. Trainings took place in July 2018 in Rwamagana, August 2018 in Rubavu and May 2019 in Kirehe. During training, community campaigns led by CHWs and nurses invited women for screening or evaluation of breast cancer symptoms. Each health centre and district hospital had a nurse focal point overseeing the services of the early detection programme in once-weekly clinics. After the training, district-level or national-level mentors visited health centres to support clinicians. The regularity of the visits was based on the availability of the mentors, but they were less regular than in the Burera pilot project.

**Fig. 1:**
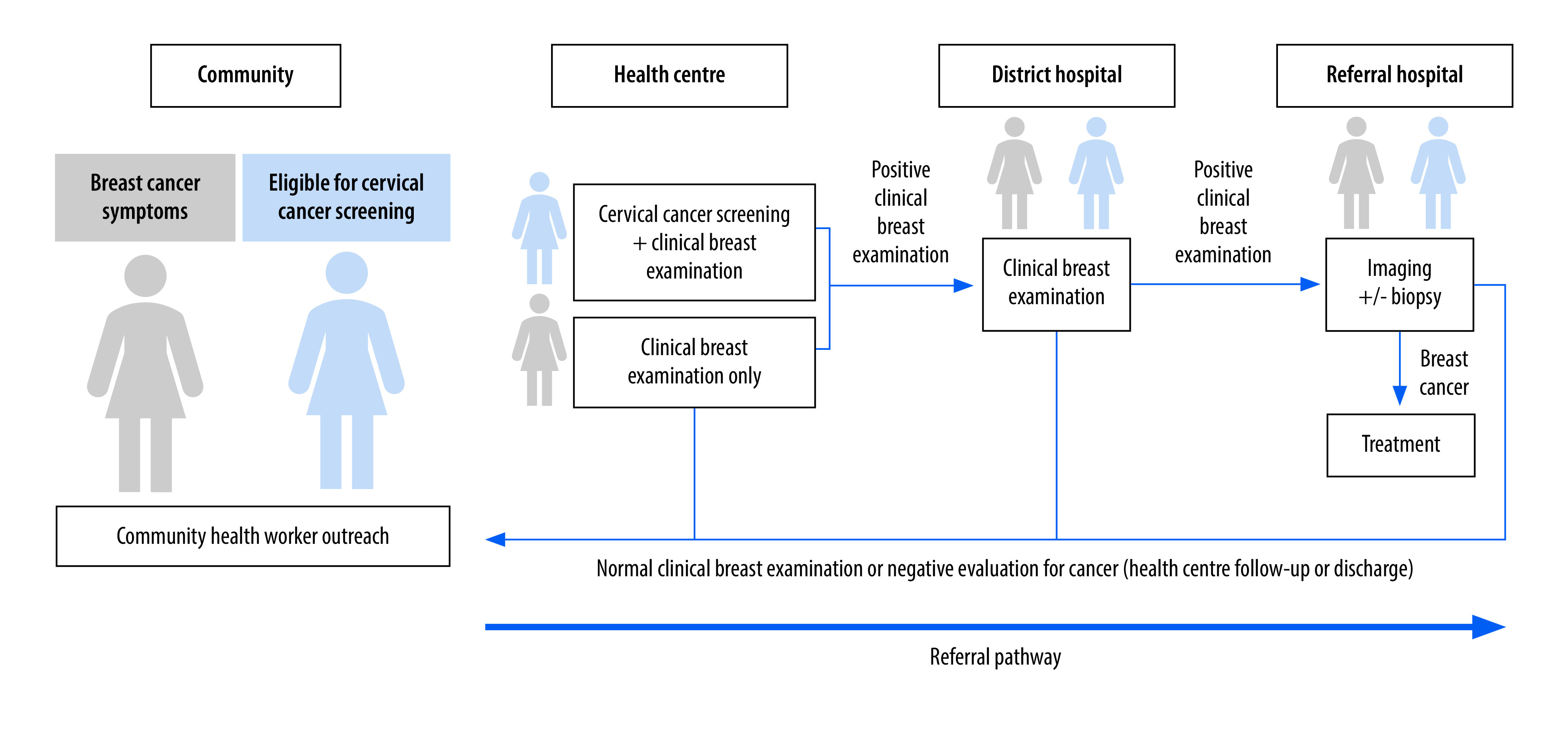
Clinical breast examination pathways in the Women’s Cancer Early Detection Program, Rwanda

At the health centres, trained nurses conducted the cervical cancer screening and/or clinical breast examination free of charge. Nurses followed standardized algorithms to determine management of women with breast concerns or abnormal breast examinations.^22^ In most cases (for example, any palpable mass, skin changes, nipple discharge or persistent pain), the guidelines instructed nurses to refer patients to district hospitals where hospital-based clinicians, either general practitioner physicians or district hospital nurses, re-assessed them. The district hospital clinicians referred women with suspicious findings to referral hospitals for diagnostic breast ultrasound and biopsy if needed (Fig. 1). Hospital-level services are not free for patients but are covered by Rwanda’s community-based health insurance,^27^ typically with a 10% co-payment. The focal points at the health centre and district hospital called a centrally located patient navigator when women were referred to a higher level of care; the navigator entered the referred women into a spreadsheet and contacted them when they missed visits. Women diagnosed with cancer were referred to the Butaro Cancer Center of Excellence for treatment. At Butaro, treatment costs for all cancer patients are subsidized by Partners In Health.^28^

The programme was funded by the Rwanda Biomedical Centre and by a grant to the University of Pennsylvania and Partners In Health from the Breast Cancer Research Foundation, a United States-based philanthropic foundation.

### Data collection and analysis

To assess implementation of the programme, we examined the frequency with which clinics within the programme were held, patient volumes and the number of referrals made. We also examined timeliness of care based on intervals between referrals and visits to the next level of care and the rates of loss-to-follow-up – non-completion of a recommended referral over the evaluation period. The primary patient outcome was breast cancer diagnosis. To evaluate whether the addition of clinical breast examination screening to cervical cancer screening led to additional cancer diagnoses, we assessed whether women diagnosed with breast cancer had initially presented for breast cancer symptoms, and whether they had first received cervical cancer screening.

Health centre focal points reported to the patient navigator whether clinics in the programme were held in Rwamagana and Rubavu between July 2018 and May 2019. We extracted the numbers of women evaluated at health centres from aggregated reports provided weekly by the focal points. We extracted individual-level data on women referred from health centres and district hospitals from the patient navigator’s tracking spreadsheet. We included all women enrolling in the programme in each district from its launch until 31 December 2019, with patient follow-up data collected until April 2021. The patient navigator documented patient telephone calls and retrospectively categorized patients’ reasons for missing visits. We gathered additional data on diagnostic intervals by reviewing the medical records in the health centres, district hospitals and referral hospitals. We determined presenting symptoms for the women diagnosed with breast cancer from health centre records, patient interviews conducted for a related qualitative study (Pace LE, et al., unpublished report, 2022), or telephone outreach. Partners In Health and Rwanda Biomedical Centre programme administrators retrospectively compiled the cost data.

### Ethical considerations

This study was approved by the Rwanda National Ethics Committee (number 98/RNEC/2021) and the Massachusetts General Brigham Institutional Review Board (number 2014P002688).

## Results

### Programme implementation

From the launch of the programme until the third week of May 2019, Rwamagana health facilities held clinics within the programme 73.9% (488/660) of the assessed weeks. Rubavu health facilities held clinics 68.4% (383/560) of the assessed weeks (Table 1 and the online repository).^29^ Over the evaluation period in the three districts, 9763 women received cervical cancer screening and clinical breast examination; 7616 additional women received clinical breast examination alone (Table 1). Of the 17 379 women in total who received clinical breast examination, 730 (4.2%) had abnormal results. Of the women with abnormal results, 585 were referred from the health centres to district hospitals for further evaluation; of these women, 436 (74.5%) attended their district hospital visit and 149 missed their visit despite follow-up by the navigator. The median interval between health centre visit and district hospital visit was 9 days (interquartile range, IQR: 3–19). A total of 200 women were referred from the district hospital to the referral hospital, 179 (89.5%) of whom attended their referral hospital visit. The median interval between district hospital visit and referral hospital visit was 11 days (IQR: 4–18).

**Table 1:** Service delivery at health centres and district hospitals participating in the Women’s Cancer Early Detection Program, by district, Rwanda, 2018–2019

Variable	District			Total
	Rwamagana (Jul 2018–Dec 2019)	Rubavu (Aug 2018–Dec 2019)	Kirehe (May–Dec 2019)	
**District population, no.**	391 826	504 578	425 460	1 321 864
**% of weeks programme clinics were held^a^**	73.9 (488/660)	68.4 (383/560)	Data not available	NA
**Health centre visits**				
Cervical cancer screening performed, no.	4302	2986	2475	9763
Clinical breast examination performed, no.	8420	5173	3786	17 379
Abnormal clinical breast examination, no. (%)	364 (4.3)	271 (5.2)	95 (2.5)	730 (4.2)
**Referral completion and timeliness**				
Referrals from health centres to district hospital, no.	278	240	67	585
Seen at district hospital, no. (%)	210 (75.5)	173 (72.1)	53 (79.1)	436 (74.5)
Days between health centre visit and district hospital visit, median (IQR)^b^	7.0 (3.0–16.0)	12.0 (3.0–20.0)	10.0 (2.0–19.5)	9.0 (3.0–19.0)
No. of referrals from district hospital to referral hospital	75	98	27	200
Seen at referral hospital, no. (%)	62 (82.7)	92 (93.9)	25 (92.6)	179 (89.5)
Days between district hospital visit and referral hospital visit, median (IQR)^c^	12.0 (6.3–20.5)	7.5 (4.0–17.8)	7.5 (5.0–14.8)	11.0 (4.0–18.0)

IQR: interquartile range; NA: not applicable.

^a^ Calculated as: number of weeks a clinic was held in any health centre/(number of weeks assessed × number of health centres). Data on the frequency of health centre and hospital clinics were available for Rwamagana and Rubavu from July 2018 until May 2019.

^b^ We excluded data on five women because of missing district hospital dates and on four women because of negative intervals (incorrect dates where the recorded hospital date was before the recorded health centre date).

^c^ We excluded data on two women because of missing district hospital dates and on one woman because of a negative interval (incorrect dates where the recorded hospital date was before the recorded health centre date).

The patient navigator was unable to determine the reason for the missed visit to the district hospital for 49.0% (73/149) of the women because no telephone number was documented in health centre records, or because the patient navigator attempted to contact the woman, but could not reach her. Of the remaining 76 women, the reasons given for not going to the district hospital included: improvement in symptoms; financial barriers, such as transport costs, insurance, or other socioeconomic issues; and being unaware of the date of their visit (online repository).^29^ Of the 21 women who did not visit the referral hospital, the patient navigator was unable to determine the reason for the missed visit for 13 (61.9%) women. For the remaining eight women, the reasons for missing the visit included improvement in symptoms, financial barriers, choosing a different health facility and seeking care from a traditional healer.

### Outcomes

A total of 29 women were diagnosed with breast cancer, 0.2% of the 17 379 who had a clinical breast examination. Of these women, 19 (65.5%) were 50 years or older (median 55; IQR: 44.0–61.0) and 23 (79.3%) had stage III or stage IV disease (Table 2). Of the 29 women with breast cancer, we were able to determine the reason for coming to the health centre in the first place for 23 women; all had sought care for breast cancer symptoms. For the remaining six women, we could not determine their reason for initial presentation from health centre records, nor was this information recorded in the referral hospital records, and they could not be reached by telephone. However, all six women had symptomatic stage III or stage IV cancer on diagnosis at the hospital. Of the women diagnosed with breast cancer, the median time between the first visit to the health centre and the first referral hospital visit was 19 days (IQR: 11–26.0; Table 2).

**Table 2:** Characteristics of women diagnosed with breast cancer through the Women’s Cancer Early Detection Program, by district, Rwanda, 2018–2019

Characteristic	District			Total
	Rwamagana	Rubavu	Kirehe	
**Diagnosed with breast cancer, no.^a^**	11	12	6	29
**Incidence per 100 000 per year^b^**	1.9	1.7	2.1	ND
**Age group in years, no. (%)**				
< 30	0	0	0	0
30–49	3 (27.3)	4 (33.3)	3 (50.0)	10 (34.5)
≥ 50	8 (72.8)	8 (66.7)	3 (50.0)	19 (65.5)
**Median age in years (IQR)**	55 (50.5–60.5)	60.5 (33.5–66.5)	50 (45.0–52.8)	55 (44.0–61.0)
**Reason for coming to the health centre, no.**				
Asymptomatic	0	0	0	0
Breast cancer symptoms	11	9	3	23
Unknown^c^	0	3	3	6
**Referral interval^d^**				
Days from first health centre visit to first referral hospital visit, median (IQR)	15.0 (11.5–54.0)	21.0 (19.0–38.0)	10.0 (6.0–18.0)	19.0 (11.0–26.0)
**Stage at diagnosis, no. (%)**				
I or II	2 (18.2)	2 (16.7)	2 (33.3)	6 (20.7)
III or IV	9 (81.8)	10 (83.3)	4 (66.6)	23 (79.3)

IQR: interquartile range; ND: not determined.

^a^ All were women.

^b^ Calculated as: no. of cases/(district population × no. of years the district was in the program in the study period). This duration was 1.5 years for Rwamagana, 1.42 years for Rubavu and 0.67 years for Kirehe.

^c^ Initial presentation was unknown if data were not recorded at the first health centre visit and the patient could not be reached for additional information.

^d^ We only included women for whom the date of their first health centre visit was known: 11 women from Rwamagana, nine from Rubavu and six from Kirehe.

### Costs

The variable, or direct costs – for example, costs of training and medical supplies – of launching the programme in 2018–2019 were US$ 78 758 (online repository);^29^ 56.1% (US$ 44 171) of this cost was covered by Partners In Health with funding from the Breast Cancer Research Foundation, and 43.9% (US$ 34 587) by the Rwanda Biomedical Centre.

## Discussion

We evaluated the integration of early detection of breast cancer within cervical cancer screening at the primary care level in a low-income country. In this early analysis at 2 years, providing a one-time clinical breast examination to all women receiving cervical cancer screening did not appear to lead to additional breast cancer diagnoses beyond those that would have been diagnosed by targeting symptomatic women. All breast cancers were diagnosed in women with symptoms at presentation. Furthermore, two thirds of the women diagnosed with breast cancer were older than the target screening age for cervical cancer.

Nevertheless, our study indicates successes of the Women’s Cancer Early Detection Program, including the establishment of breast cancer and cervical cancer screening within the public health-care system for a population without previous access to these services. Clinics of the programme were held at health centres and district hospitals most weeks in the first 10–11 months of the programme. Of the women who received hospital care, the time between appointments was short, suggesting effective referral pathways that meet WHO’s recommended interval of 60 days between care-seeking and diagnosis.^16^ The programme was led by the government in response to Rwanda’s national cancer control plan, and implemented in health facilities through multi-institutional partnerships, which demonstrates the potential for coordinated, sustained impact. In interviews, reported separately in published and unpublished reports,^30^ both clinicians and patients described increased community and clinician awareness, strong support for the programme and improved clinician skills.

However, the initial approach of the early detection programme had some drawbacks. First, in addition to the limited breast cancer detection among asymptomatic women receiving cervical cancer screening, the cancer diagnosis rate in the districts covered by the programme (1.7–2.1 cases per 100 000 population per year) was markedly lower than would be expected based on the estimated breast cancer incidence in Rwanda (about 9.5 cases per 100 000 in 2020).^31^ This finding suggests that many women with incident cancer did not seek services from the programme during our evaluation period. The incidence was also lower than the breast cancer incidence rate for the pilot intervention in Burera district (6.9 cases per 100 000 per year).^23^ This finding could partly be because the programme primarily targeted the relatively young population eligible for cervical cancer screening. Although the low detection rates could reflect the limited sensitivity of clinical breast examination, they also suggest the potential benefit and efficiency of intensified focus on women with breast cancer symptoms, as well as on others at higher risk of cancer, such as women with a family history of breast cancer.

Second, the high proportion of late-stage diagnoses was concerning. This finding may reflect prevalent undiagnosed disease in this previously unscreened population, or could reflect limitations of health centre clinicians’ clinical breast examinations for detection of early-stage, smaller tumours. However, the positivity rate of clinical breast examinations through the programme was comparable or higher than in clinical breast examination studies in other low- and middle-income countries.^7^^,^^8^^,^^32^^,^^33^ Furthermore, in written tests and observations, we found substantial improvement in the skills of the clinicians working in the early detection programme immediately after training. However, the programme lacked resources to implement the Burera pilot programme’s frequent clinical mentorship,^34^ and skill sustainment was not rigorously assessed. The lack of early-stage diagnoses could also reflect limited care-seeking by women with symptoms of early-stage cancer. This finding highlights the need for intensified case-seeking and community engagement.

Third, despite engagement of a patient navigator, a quarter of the women referred to district hospitals did not attend their appointments. This rate is similar to cancer screening programmes in Sudan,^9^ although lower than the loss-to-follow-up rates in a large-scale programme in India.^8^ Even though the patient navigator tried to contact the women who missed their appointments, she was unable to reach about half of the women because they did not have documented telephone numbers or were unavailable. Team members also noted that the patient navigator had to spend a substantial amount of time entering data, and hence had limited time for follow-up of the women. In interviews, patients and clinicians mentioned poverty and transportation costs as important barriers to attending referral appointments (unpublished and published data).^30^ However, funds were not routinely available to allow the patient navigator to support patient transportation and other costs. These findings highlight the need for resources to support patients to complete the referral process.

These early findings from the early detection programme have led to adaptations to the programme. First, given the limited added benefit of clinical breast examination screening at this stage, the Rwanda Biomedical Centre is implementing a modified programme in eight additional districts to direct focus on individuals with breast cancer symptoms, which is in line with WHO recommendations for early diagnosis of cancer.^14^ In this modified programme, women receiving cervical cancer screening are asked about breast concerns and are given a clinical breast examination only if they have concerns, or request one. Evaluation is underway to examine the effectiveness and efficiency of this approach. Ongoing efforts to decentralize diagnostic services, such as providing ultrasound at district hospitals, may reduce barriers to adherence to diagnostic evaluation. National cancer control initiatives should also include systematic strategies to monitor and improve the quality of clinical breast examination, such as the more structured mentorship programme in Burera. Second, to address challenges with patient loss-to-follow-up, Rwanda Biomedical Centre has overseen the development of a tablet-based electronic medical record specific to the early detection programme, to facilitate documentation of care and patient retention. Finally, evolving policies and practices in the care of older women in Rwanda and globally may facilitate early detection of breast cancer in this at-risk population. For example, Rwanda has now adopted WHO’s expanded cervical cancer screening recommendations to unscreened women aged 50–65 years;^35^ these women may be important candidates for breast cancer screening.

Our study has some limitations. First, this analysis captures only the initial phase of the early detection programme. Outcomes such as stage distribution will change as breast cancer awareness improves and incident cancers are detected more than prevalent cancers. In addition, educating women receiving cervical cancer screening about breast cancer will likely have long-term benefits by increasing breast health awareness in younger women,^36^ an outcome not captured by our short-term analysis. Second, because we only had aggregated data on the women presenting at health centres who were not referred, we do not know their ages, sex or the reasons for seeking a clinical breast examination. Third, because of resource constraints, our evaluation primarily focused on breast care and outcomes. Future analyses should examine the effect of adding clinical breast examination on cervical cancer screening reach and quality. Finally, our costing analysis was not prospectively planned and only included variable costs. While knowledge of these costs is valuable for programme planning, inclusion of fixed costs such as clinical staff salaries, as captured in the Burera costing analysis,^24^ will better reflect the programme’s true costs.

While Rwanda’s Women’s Cancer Early Detection Program succeeded in establishing women’s cancer screening services with cervical cancer screening, the initial benefit in diagnosing additional breast cancer is limited, probably because of the screening approach and the relatively young population. For resource-constrained health systems seeking to facilitate early detection of breast cancer, an initial focus on women with breast cancer symptoms may allow facilities to improve service delivery, efficiency and quality as early diagnosis capacity grows. For example, efforts are needed to increase awareness of early breast cancer symptoms among women and ensure links to timely diagnosis and treatment for those with cancer. Decentralization of certain diagnostics, enhanced patient navigation and support with transport costs could reduce follow-up barriers. In addition, systems are needed to assure the quality of clinical breast examination. Once strong early diagnosis systems are in place, a breast cancer screening model might increase reach and impact, although research will be needed to determine the best age groups to screen. Longer-term analyses of clinical and implementation outcomes (including costs) will help strengthen strategies to improve outcomes for the growing number of women with breast cancer in Rwanda and sub-Saharan Africa.
